# Review of the Capitellidae (Annelida, Polychaeta) from the Eastern Tropical Pacific region, with notes on selected species

**DOI:** 10.3897/zookeys.151.1964

**Published:** 2011-12-03

**Authors:** María Elena García-Garza, Jesús Angel De León-González

**Affiliations:** 1 Laboratorio de Biosistemática, Facultad de Ciencias Biológicas, Universidad Autónoma de Nuevo León, Apartatado Postal 5 ‘F', San Nicolás de los Garza, Nuevo León, México

**Keywords:** Capitellidae, Catalogue, taxonomy, Eastern Tropical Pacific

## Abstract

The main objective of this work is to contribute to the taxonomic knowledge of the species of Capitellidae reported for the Eastern Tropical Pacific. This catalogue includes the original name of each species, new names, synonymies, type localities, the museum or institution where the type material is deposited, revision of the material reported for the region by different authors, new examined material, previous reports from other regions of the world, and comments on systematics and distributions. The catalogue lists 43 species in 19 genera. Of these, 6 species were erroneously recorded for the region (*Decamastus gracilis* Hartman, 1963; *Decamastus nudus* Thomassin, 1970; *Mastobranchus variabilis* Edwing, 1984; *Notomastus aberans* Day, 1957; *Notomastus americanus* Day, 1973; *Notomastus latericeus* Sars, 1851) and 5 species are found here to be questionable records for the Eastern Tropical Pacific (*Capitella capitata* (Fabricius, 1780); *Dasybranchus glabrus* Moore, 1909; *Decamastus lumbricoides* Grube, 1878; *Notomastus lineatus* Claparède, 1870 and *Notomastus tenuis* Moore, 1909).

## Introduction

Capitellids are typical inhabitants of marine soft bottom sediments. They are important in the energy budgets of the environments due to their feeding habits and usually high abundances. Their morphology is very simple; most of the species are similar morphologically to earthworms. Generally they live buried in sand or mud and feed by assimilating the organic matter adhering to sediments and are thus regarded as non-selective deposit feeders (Dean 2001). Capitellids are distributed from the intertidal zone to the deep sea and are often dominant components of the infaunal community, especially in organically enriched sediments. For their importance as indicators of changes in the ecosystems, the capitellids have been the subject of numerous ecological studies. However, there are only a few taxonomic publications in which their morphological variations have been analyzed. For this reason there has been a great number of errors made in the identification of species and many species distributions are considered doubtful.

The taxonomy of the Capitellidae is based almost entirely on the distribution, orientation, and structure of chaetae for identification at all levels (Doyle 1991). The recognition of the genera in this family has been based traditionally on the chaetal formula, in other words, the number of segments with capillary chaetae and, if present, the mixture of capillary chaetae and hooded hooks (Fauchald 1977, Amaral 1980). Although there are certain ontogenetic variations, the chaetal formula is useful in identifing adult stages.

The capitellids of the Eastern Tropical Pacific have been studied by numerous authors included in the checklist of Salazar-Vallejo and Londoño Mesa (2004). In addition the capitellids of the Pacific in Costa Rica have been studied by Dean (2001).

## Methods

In first instance the list of species was taken from a checklist of polychaetes of the Tropical Oriental Pacific (Salazar-Vallejo and Londoño-Meza 2004), and other published literature. Whenever possible, the species included in collections of different institutions previously reported for the region were revised. Genera and species are listed in alphabetical order, and the following information was provided for each species: name, authorship and date of publication, synonymy, type material, material examined from the region (most of it collected and analyzed by the authors), records, and Remarks. The abbreviations of institutions where type material may be found are given.

Abbreviations of the institutions cited in the catalogue:

BMNH The Natural History Museum, London, UK

CAS-IZ California Academy of Sciences-Invertebrate Zoology, San Francisco, USA

ECOSUR El Colegio de la Frontera Sur Chetumal, México

CP ICMyL UNAM Polychaete Collection of the Instituto de Ciencias del Mar y Limnología, Universidad Nacional Autónoma de México, México

LACM-AHF Natural History Museum of Los Angeles County, Los Angeles, USA

MNHN Muséum National d'Histoire Naturelle, Paris, France

UANL Universidad Autónoma de Nuevo León, México

USNM National Museum of Natural History, Washington, DC, USA

HZM Zoologisches Institut und Zoologisches Museum der Universität, Hamburg

ZMUC Zoological Museum, University of Copenhagen

## Results

### Taxonomy

**Class Polychaeta Grube, 1850**

**Family Capitellidae Grube, 1862**

#### Genus Amastigos

Piltz, 1977

Amastigos Piltz, 1977: 57, figs 1–2.

##### Type species.

*Amastigos acutus*Piltz, 1977

##### Amastigos acutus

Piltz, 1977

http://species-id.net/wiki/Amastigos_acutus

Amastigos acutus Piltz, 1977: 57, figs 1–2.

###### Type locality.

Near Carpinteria Hope Ranch, CaliforniaUSA, Stn. 11A, April 5 1971, fine grained, sandy beach.

###### Type material.

Holotype (LACM-AHF POLY 1239) and 1 Paratype (LACM-AHF POLY 1240).

###### Material examined.

(4 specimens) Baja California Sur: La Paz Bay, Ensenada de La Paz (UANL-6457), Stn. 1 [24°06'50.3"N, 110°25'12.0"W] (1 specimen), August 17 2005; (UANL-6459), Stn. 7 [24°09'00.0"N, 110°21'38.0"W] (1 specimen) November 27 2005; (UANL-6456), Stn. 11 [24°08'28.9"N, 110°25'41.9"W] (1 specimen), November 27 2005, 0.20 m, Coll. Daniel Hernández-Valdez (DHV), Guerrero: Petacalco Bay (UANL-6458), Stn. E-2 [17°58'26"N, 102°03'45"W] (1 specimen), december 9 1992, 7 m, Coll. Edgar Amador-Silva.

###### Records.

USA: California (Piltz 1977); México: Baja California Sur, La Paz Bay; Guerrero, Petacalco Bay (material reported in this work).

###### Remarks.

Upon examining the holotype, we noticed some morphological characteristics that were omitted in the original description: a) the presence of palpodes on the anterior end of the prostomium, and b) the structure described as the pygidium is, in fact, the rupture of an abdominal segment. The rest of the description agrees well with the features of the type material

###### .

#### Genus Anotomastus

Hartman, 1947

Anotomastus Hartman, 1947: 441, Pl. 9, fig. 56.

##### Type species.

*Eunotomastus gordiodes* Moore, 1909

##### Anotomastus gordiodes

(Moore, 1909)

http://species-id.net/wiki/Anotomastus_gordiodes

Eunotomastus gordiodes Moore, 1909: 278, Pl. 9, fig. 56.
Anotomastus gordiodes Hartman, 1947: 442, Pl. 58, figs 1–6; Reish 1968: 89; Hartma 1969: 355.

###### Type locality.

San Diego, California.

###### Type material.

Syntypes (LACM-AHF POLY 1450-42), (LACM-AHF POLY 1451-42), (LACM-AHF POLY 1505-43), (LACM-AHF POLY 1487-38), (LACM-AHF POLY 2275).

###### Material examined.

(50 specimens) Baja California: Todos Santos Bay (ECOSUR-CAP-1), Stn. IV-2 [28°56'32.3"N, 113°33'15.4"W] (1 specimen), 13 m, October 8 1979, Coll. Sergio Salazar-Vallejo (SSV); Los Angeles Bay, Municipal Beach (UANL-6463) [28°56'32.3"N, 113°33'15.4"W] (26 specimens), 1 m, June 27 2005; Baja California Sur: Magdalena Bay (UANL-6245), Stn. E-9 [25°36'8"N, 112°0'8"W] (1 specimen), December 6 1996; (UANL-6246), Stn. E-2 [24°34'9"N, 111°58'9"W] (2 specimens), December 5 1996; 1 m, Coll. Victoria Díaz-Castañeda (VDC); Sonora: Puerto Peñasco, La Choya Bay (UANL-6462) [31°20'37.8"N, 113°38'01.7"W] (16 specimens) 1 m, June 29 2005; (UANL-6464) (4 specimens), 1 m, August 7 2006, Coll. Jesús Angel de León-González (JALG) and María Elena García-Garza (MEGG).

###### Records.

USA: California (Moore 1909, Hartman 1947, 1969); México: Baja California, Los Angeles Bay (Reish 1968), Baja California, Gulf of California, Baja California Sur and Sonora in the Gulf of California (present study).

#### Genus Capitella

Blainville, 1828

Capitella Blainville, 1828:368.

##### Type species.

*Lumbricus capitatus* Fabricius, 1780:279.

##### Capitella capitata

(Fabricius, 1780)

http://species-id.net/wiki/Capitella_capitata

Capitella capitata capitata Warren, 1976:198, pl. 1.
Capitella capitata Fabricius, 1780: 279; Fauvel 1927: 154, fig.155; Reish 1963: 428; 1968: 89; Day 1967: 595, fig. 28.2 i–m; Hartman 1969: 361, figs 1–5; Bastida-Zavala 1993: 22; Blake 2000: 58, fig.4.2 a–e; Dean 2001: 71; 2004: 135; Blake et al. 2009:58, figs 2 a–f, 3 a–f, 4 a–f.

###### Type locality.

Greenland, NW Atlantic, Arctic Ocean

###### Type material.

Neotype ZMUC- POL-1967 (not seen).

###### Material examined.

(181 specimens) Baja California Sur: La Paz Bay, Magdalena Bay [24°38'09"N, 112°80'73"W] (139 specimens), December 6 1996, Coll. JALG; Sonora: Guaymas Bay (UANL 5238), Stn. 2 [27°53'28"N, 110°54'14"W] (18 specimens), May 6 1999; (UANL 5241), Stn. 6 [27°55'09"N, 110°52'39"W] (4 specimens), May 4 1999; (UANL 5242), Stn. 3 [27°54'13"N, 110°53'59"W] (2 specimens), May 6 1999; (UANL 5244), Stn. 1 [27°55'21"N, 110°53'14"W] (1 specimen), May 6 1999; Río Escondido [27°54'04.2"N, 110°54'08.2"W] (17 specimens), August 9 2006, 0.50 m, Coll. JALG.

###### Records.

This species has been reported to be broadly distributed around the world: Greenland (Blake 2009), France (Fauvel 1927), Southern Africa (Day 1967), USA, California (Hartman 1969, Blake et al. 2000), Costa Rica (Dean 2001, 2004), Western México (Reish 1963, 1968, Bastida-Zavala 1993). Blake (2009) indicates that *Capitella capitata* is probably only distributed in Arctic and subarctic areas and those numerous records from lower latitudes are probably not this species.

###### Remarks.

The specimens revised in this work from Western México are similar morphologically to the neotype of *Capitella capitata* redescribed by Blake (2009). The type locality (Greenland) of this species could indicates that records from warmer waters must be considered doubtful so that molecular studies may be required to separate them from the true "*Capitella capitata*" populations.

#### Genus Dasybranchethus

Monro, 1931

Dasybranchethus Monro, 1931:26

##### Type species.

*Dasybranchethus fauveli* Monro, 1931

##### Dasybranchethus pacifica

García-Garza & de León-González, 2009

http://species-id.net/wiki/Dasybranchethus_pacifica

Dasybranchethus pacifica García-Garza & de León-González, 2009: 1437 fig. 1A-D.

###### Type locality.

Baja California Sur, Concepción Bay, El Quemadito Beach [26°45'33.1"N, 111°52'36.5"W], June 26 2005; Santispac mangrove [26°45'43.2"N, 111°53'31.0"W], 1 m, June 26 2005, Coll. MEGG and JALG. Silt with a mixture of thick and fine sand, intertidal.

###### Type material.

Holotype (UANL-6336) and 1 Paratype (UANL-6337)

###### Records.

México: Baja California Sur, Concepción Bay.

#### Genus Dasybranchus

Grube, 1850

Dasymallus Grube, 1846:45. (Junior homonym of the Coleoptera genus *Dasymallus* Dejean, 1835)
Branchoscolex Schmarda, 1861:164.

##### Type species.

*Dasybranchus caducus* (Grube, 1846)

##### Dasybranchus caducus

(Grube, 1846)

http://species-id.net/wiki/Dasybranchus_caducus

Dasybranchus caducus Monro, 1928:97; Monro 1933: 1039; Fauvel 1943: 26; Rioja 1947: 523.

###### Type locality.

Mediterranean Sea.

###### Type material.

Not seen

###### Material examined.

Not seen

###### Records.

México, Baja California Sur, Gulf of California, La Paz Bay, El Mogote Beach (Rioja 1947); Lagoon of the San José Island (Fauvel 1943); Panamá (Monro 1928) Galápagos Islands (Monro 1933).

###### Remarks.

The material examined by Fauvel (1943) was not found in the MNHN, and the specimens reported for México by Rioja (1947) are lost (Salazar-Vallejo 1989). In both cases Fauvel and Rioja, did not provide a complete description or illustrations of the morphological structures.

##### Dasybranchus glabrus

Moore, 1909

http://species-id.net/wiki/Dasybranchus_glabrus

Dasybranchus glabrus Moore, 1909: 280; Treadwell 1941: 212; Hartman 1947: 434; 1969; Bastida-Zavala 1993: 22; Rioja 1941: 730

###### Type locality.

California, San Diego Bay

###### Type material.

Not seen

###### Material examined.

(2 specimens)Baja California Sur: La Paz Bay, Enfermería Beach, Stn. E-19 [24°13'54.4"N, 110°18'22.3"W] (2 specimens), December 1985, mixed silt, sands and fragmented shells.

###### Records.

USA: California (Moore 1909, Treadwell 1941, Hartman 1947). México: Cedros Island (Hartman 1947), Acapulco Rioja (1941).

###### Remarks.

*Dasybranchus glabrus* was described from California, and recorded by Bastida-Zavala (1993) from La Paz Bay, Baja California Sur. However, upon examination of those materials we noticed that the specimens were morphologically similar to the holotype of *Dasybranchus parplatyceps* Kudenov 1975. Then, the record of *Dasybranchus glabrus* from Acapulco, México should not be considered valid because it was based on an incomplete specimen its diagnosis is brief and lacks the important features for unequivocal identification.

##### Dasybranchus lumbricoides

Grube, 1878

http://species-id.net/wiki/Dasybranchus_lumbricoides

Dasybranchus caducus var. *lumbricoides* Monro, 1933:1059; Berkeley and Berkeley 1941: 49.
Dasybranchus lumbricoides Grube, 1878: 190:40; Hartman 1947:431(*partim*); Fauchald 1972: 241; 1977: 52; Laverde-Castillo 1986: 125; Molina-Lara and Vargas-Zamora 1995: 198; López et al. 1997: 66; Dean 2001: 73; 2004: 135; Rivera and Romero 2008: 19.

###### Type locality.

Philippine Islands

###### Type material.

Not seen

###### Material examined.

Baja California Sur: 3.4 miles S W of Punta Arena, Carmen Island, Stn. 1746 [25°46'00"N, 111°15'00"W, to 25°49'40"N, 111°15'30"W] (1 specimen), 29-35 m, March 18 1949, sand, mud, pebbles. Coll. Allan Hancock Foundation Velero IV (AHF); California, Corona del Mar, New Port, Harbor, Stn. 1450-42 [33°36'04"N, 117°52'48"W] (1 specimen), 1.51 m; Stn. 1451-42, [33°36'04"N, 117°52'48"W] (1 specimen), 1.2 m.

###### Records.

California (Monro 1933, Berkeley and Berkeley 1941, Hartman 1947). México, Tiburón Island (Hartman 1947), México, Carmen Island (Fauchald 1972) Panamá, Canal de Panamá, Gorgona Island (Fauchald 1977), Coiba Island (López et al. 1997); Colombia, Gorgona Island (Laverde-Castillo 1986); El Salvador (Molina-Lara and Vargas-Zamora 1995; Rivera and Romero 2008); Costa Rica: Gulf of Nicoya (Dean 2001, 2004).

###### Remarks.

*Dasybranchus lumbricoides* was described from the Philippine Islands and Fauchald (1972) reported it for Carmen Island in the Gulf of California. Upon examination of Fauchald's specimens it was determined that they were actually *Notodasus harrisae* García-Garza et al. 2009. This was also the case for material identified as *Dasybranchus lumbricoides* by Hartman (1947) from Corona del Mar, Newport Harbor The record of Hartman 1947 from Tiburón Island was not available for examination and we believe that her identification should also be considered doubtful.

##### Dasybranchus parplatyceps

Kudenov, 1975

http://species-id.net/wiki/Dasybranchus_parplatyceps

Dasybranchus parplatyceps Kudenov, 1975: 218, figs 31–34; Hernández-Alcántara and Solís-Weiss 2003: 4.
Notomastus (Clistomastus) lineatus fide Bastida–Zavala 1993: 22.
Dasybranchus glabrus fide Bastida–Zavala, 1993: 22.
Decamastus gracilis fide Bastida–Zavala, 1995: 12.
Leiocapitella glabra fide Bastida–Zavala, 1995:12.

###### Type locality.

Sonora, Puerto Peñasco, Stn. Beach [31°16'40"N, 113°30'W]. Intertidal, in silt under basalt boulders (Holotype, January 21 1972, Paratype, April 16 1972), Coll. J.D. Kudenov.

###### Type material.

Holotype (LACM-AHFPOLY 1111) and 1 Paratype (LACM-AHF POLY 1112).

###### Material examined.

(120 specimens) Baja California: Los Angeles Bay, Moradas Beach [28°57'10.8"N, 113°33'27.0"W] (1 specimen), May 25 1986; (2 specimens), August 23 1987 Coll. JALG and SSV; La Gringa Beach (UANL-6431) [29°02'17.0"N, 113°32'57.2"W] (2 specimens), 1 m, June 27 2005; (UANL-6429) (2 specimens), 2 m, August 4 2006, Col. JALG and MEGG; Baja California Sur: La Paz Bay, Caimancito Beach, Stn. 1 [24°12'37"N, 110°18'19"W] (5 specimens), 1 m, March 19 1989; Stn. C-33 [24°13'55.6"N, 110°18'23.7"W] (2 specimens), 1.5 m, November 29 1986; Stn. C-37 [24°11'35.6"N, 110°18'00.7"W] (9 specimens), 1.5 m, November 29 1986; Enfermería mangrove, Stn. E-19 [24°13'54.4"N, 110°18'22.3"W] (2 specimens), 1 m March 1986, Coll. JALG; Cabo Pulmo-Los Frailes, Stn. CP-988-2 [23°24'27"N, 109°24'26"W] (5 specimens), 4-7 m, September 18 1988, Coll. JRB-Z; Enfermería Beach [24°13'55.6"N, 110°18'23.7"W] (2 specimens), 1m, June 15 1987; El Saladito Beach (2 specimens), 1m, May 15 1987; El Presidente Beach (2 specimens), October 8 1987; (2 specimens ), 1m, October 10 1987, Coll. JALG & SISV; Concepción Bay, El Requesón Beach (UANL-6430) [26°38'17.7"N, 111°49'55.5"W] (1 specimen), July 19 1985; (UANL-6438) (9 specimens) 1m, August 20 1985, Coll. JALG; El Quemadito Beach (UANL-6433) [26°45'33.1"N, 111°52'36.5"W) (1 specimen), 1m, June 25 2005; Los Cocos Beach (UANL-6437) [25°44'39.1"N, 111°53'55.4"W] (3 specimens), 1 m, June 25 2005, Coll. JALG and MEGG; Magdalena Bay Estero Rancho Nuevo, Santa Marina Bay, (UANL-6428) [24°19'15"N, 111°25'05"W] (6 specimens), 3 m, June 15 1998; Western coast of Baja California Peninsula, San Juaníco Bay (UANL-6439) [26°02'12"N, 112°51'12"W] (2 specimens), January 21 1999, 30 m, Coll. JALG; Sonora: Guaymas, Varadero Beach (UANL-6427) [27°54'04.3"N, 110°52'07.7"W] (11 specimens), 1 m, July 1 2005, Coll. JALG and MEGG; Nayarit: María Madre Islands (UANL-6463) [21°38'14"N, 116°32'13.7"W] (1 specimen), 3 m, November 2 1979, Coll. SS-V.

###### Records.

México: Sonora, Station Beach, Puerto Peñasco (Kudenov 1975). Baja California Sur, continental shelf (Hernández-Alcántara and Solís-Weiss 2003). Baja California Sur (Bastida-Zavala 1993, 1995), Baja California Sur, Sonora and Nayarit (present study).

###### Remarks.

Upon careful examination the specimens reported by Bastida-Zavala (1993 and 1995) as *Notomastus (Clistomastus) lineatus*, *Dasybranchus glabrus*, *Decamastus gracilis* and *Leiocapitella glabra* were all found, to correspond to *Dasybranchus parplatyceps* Kudenov, 1975.

##### Dasybranchus platyceps

Hartman, 1947

http://species-id.net/wiki/Dasybranchus_platyceps

Dasybranchus platyceps Hartman, 1947: 435, Pl. 55, figs 1-5; Kudenov 1973: 76.

###### Type locality.

Punta Cholla, Sonora, Gulf of California, May 9 1941, intertidal.

###### Type material.

Holotype(LACM-AHFPOLY 0431) and 2 Paratypes (LACM-AHFPOLY 0432).

###### Records.

México: Punta Cholla, Sonora, Gulf of California (Hartman 1947, Kudenov 1973).

**Genus *Decamastus* Hartman, 1963**

##### Decamastus gracilis

Hartman, 1963

http://species-id.net/wiki/Decamastus_gracilis

Decamastus gracilis Hartman, 1963: 27; 1969:375; Bastida-Zavala 1995: 12. Blake 2000: 63.

###### Type locality.

California, Redondo Canyon, south wall, 1.65 miles WSW of end of Redondo Bach Pier [33°49'42"N, 118°25'18"W], December 5 1952, Coll. Allan Hancock Foundation R/V Velero IV, 232 m, green mud, fine sand

###### Type material.

Holotype(LACM-AHFPOLY 000435) and 1 Paratype (LACM-AHFPOLY 000436).

###### Material examined.

Baja California Sur, La Paz Bay, Sta.989-2 (1 specimen), September 18, 1989, 4-17 m, Coll. Rolando Bastida-Zavala.

###### Records.

USA: California (Hartman 1963).

###### Remarks.

*Decamastus gracilis* was described from Southern California (USA) and Bastida-Zavala (1995) reported it from La Paz Bay. After examination of Bastida-Zavala's materials we conclude that they correspond to *Dasybranchus parplatyceps* and that the record of *Dasybranchus gracilis* from México is erroneous.

##### Decamastus nudus

Thomassin, 1970

http://species-id.net/wiki/Decamastus_nudus

Decamastus nudus Thomassin, 1970: 81; Hernández-Alcántara and Solís-Weiss 1993: 1033; Hernández-Alcántara and Solís-Weiss 1998: 709; Hernández-Alcántara and Solís-Weiss 1999: 27; Dean 2001: 73; 2004: 136.

###### Type locality.

S.W. of Madagascar, in Tuléar region.

###### Type material.

Not seen

###### Material examined.

(21 specimens) Baja California: Rocks Consag, Stn. 39 [30°59.4'N, 114°04.1'W] (1 specimen), 106.4 m, silty sand, March 16 1985; Sonora: North Tiburón Island, Sta. 26 [29°23.3'N, 112°30.7'W] (7 specimens), 71.9 m, fine sand, March 14 1985; Cabo San Miguel, Sta. 20 [28°08.0'N, 112°45.8'W] (10 specimens), 54.1 m, fine sand, March 13 1985; Santa María Bay,Stn. 3 [25°02.4'N, 108°31.7'W] (3specimens), 32 m, March 10 1985; Punta San Marcial, Sta. 10 [25°58.6'N, 111°06.9'W] (7 specimens), 39 m, March 11 1985, Coll. PH-A & VS-W

###### Records.

Madagascar (Thomassin 1970), Costa Rica (Dean 2001; 2004)

###### Remarks.

*Decamastus nudus*, described from Madagascar, was reported by Hernández-Alcánatra and Solís-Weiss (1993, 1998, 1999) for the Gulf of California, but these specimens differ from the original description of Thomassin (1970), in having prostomium with palpode, dark brown eyespots, epithelium smooth on first segments, and hooded hooks with a main fang with four rows of small teeth. *Decamastus nudus* lacks a palpode, has orange pigment spots, the first 3–4 segments are slightly tessellated, and hooded hooks with a main fang and 2–3 rows of small teeth. We consider that these specimens belong to a different species not previously described. For that the record of *Decamastus nudus* from Gulf of California is erroneous.

#### Genus Heteromastus

Eisig, 1887

Ancistria Quatrefages, 1865
?Areniella Verrill, 1874
Heteromastus Eisig, 1887: 835.

##### Type species.

*Capitella filiformis* Claparède, 1864 by subsequent designation by Eisig (1887).

##### Heteromastus filiformis

(Claparède, 1864)

http://species-id.net/wiki/Heteromastus_filiformis

Capitella filiformis Claparède, 1864: 49, Pl. 4, fig. 10; Eisig 1887: 841.
Heteromastus filiformis Eisig, 1887: 839; Fauvel 1927: 150, fig. 53 a-i; Hartman 1947: 427, Pl. 52, figs 1–4; Wesenberg-Lund 1949: 339; Ushakov 1965: 304; Day 1967: 601; Hartman 1969: 377, figs 1–5; Hartmann-Schröder 1971: 400; Hutchings and Rainer 1979: 778; 1981: 373; Blake 2000: 69 fig. 4.8 a–g; Dean 2001: 75; 2004: 136; Green 2002: 267.

###### Type locality.

Port-Vendres France, Mediterranean Sea.

###### Type material.

Not found

###### Material examined.

(19 specimens). Mazatlán Sinaloa, Estero de Urías (EMU-418) [23°12'N, 106°23'W] (19 specimens), 0 m, January 1979, Coll. Agnes Rutgers (AR).

###### Records.

Italy (Eisig 1887), France, Mediterranean coast (Claparède 1864), France, Atlantic coast (Fauvel 1927), California, Florida, North Carolina and Massachusetts (Hartman 1947), Iranian Gulf (Wesenberg-Lund 1949), Japan Sea and Okhotsk Sea (Ushakov 1965), Southern Africa (Day 1967), California (Hartman 1969, Blake 2000), Germany (Hartmann-Schröder 1971), Australia (Hutchings and Rainer 1979, 1981), Costa Rica (Dean 2001, 2004); Thailand (Green 2002). México, Sinaloa (present study).

#### Genus Leiocapitella

Hartman, 1947

Leiocapitella Hartman, 1947: 437

##### Type species.

*Leiocapitella glabra* Hartman, 1947

##### Leiocapitella glabra

Hartman, 1947

http://species-id.net/wiki/Leiocapitella_glabra

Leiocapitella glabra Hartman, 1947: 438, Pl. 54, figs 1–3; Bastida-Zavala 1995: 12; Hernández-Alcántara and Solís Weiss 1998: 709; 1999: 27.

###### Type locality.

Holotype, 1 mile northwest of San Gabriel Bay, Espíritu Santo Island, Gulf of California, México, Stn. 1107–40 [24°26'39"N, 110°22'53"W to 24°26'19"N, 110°22'45"W], 8–10 m, February 13 1940, Allan Hancock Pacific Expedition of 1940, Velero III; Paratype, 2 miles southwest of Cedros Islands light, Stn. 1265–41 [28°20'33"N, 115°10'10"W to 28°20'45'N, 115°09'45"W], 16 m, February 28 1941, Allan Hancock Pacific Expedition of 1941, Velero III, in soft bottom.

###### Type material.

Holotype (LACM-AHF POLY 0425) and 1 Paratype (LACM-AHF POLY 0205).

###### Material examined.

(8 specimens)Baja California Sur:Cabo Pulmo-Los Frailes, Stn. CP-988 [23°24'27"N, 109°24'26"W] (8 specimens), 4-7 m May 7–8 1989, Coll. JRB-Z.

###### Records.

México:Baja California Sur, Espiritu Santo Island and Cedros Island (Hartman 1947).

###### Remarks.

After examination of the specimens reported by Bastida-Zavala (1995) as *Leiocapitella glabra* it was found that this corresponds to *Dasybranchus parplatyceps* Hartman 1947. Therefore this species is only known from the original description locality in the Gulf of California.

#### Genus Leiochrides

Augener, 1914

Leiochrides Augener, 1914:437.

##### Type species.

*Leiochrides australis* Augener, 1914

##### Leiochrides hemipodus

Hartman, 1960

http://species-id.net/wiki/Leiochrides_hemipodus

Leiochrides hemipodus Hartman, 1960: 136; Hartman 1969: 381, fig. 1; Fauchald 1972: 242; Hernández-Alcántara and Solís-Weiss 1998: 709; 1999: 27; Blake 2000: 71 fig. 4.9 a–e.
Leiochrides sp. Hartman, 1963: 62.

###### Type locality.

7.5 miles north of west end of Santa Catalina Island, California, bearing 002, Sta. 2798–54 [33°36'00"N, 118°36'03"W] 117 m, May 25 1954, Allan Hancock Pacific Expedition of 1940. Velero IV, blue green mud.

###### Type material.

Holotype **(**LACM-AHF POLY 0429) and 1 Paratype **(**LACM-AHF POLY 0430).

###### Material examined.

(3 specimens)Baja California Sur: Cabo Falso, Stn. 13775 [22°34'15"N, 109°32'30"W] (1 specimen), 441.96 m, January 22 1970; Jalisco: Cabo Corrientes, Stn. 13753 [19°41'15"N, 105°53'00"W] (1 specimen), 381 m, January 18, 1970; Stn. 13756 [19°51'00"N, 105°57'30"W] (1 specimen),426.72 m, January 18 1970, Coll. Allan Hancock Pacific Expedition of 1940. Velero IV.

###### Records.

USA: California (Hartman 1960, 1969), Santa Maria Basin (Blake 2000); Baja California Sur and Jalisco (Fauchald 1972, present study), Gulf of California (Hernández-Alcántara and Solís Weiss 1999).

##### Leiochridescf. pallidor

(Chamberlin, 1918)

http://species-id.net/wiki/Leiochrides_pallidor

Notomastus pallidor Chamberlin, 1918: 179; Berkeley and Berkeley 1942: 198.
Leiochrides pallidor Hartman, 1947: 429; 1969: 382.
Leiochrides cf. *pallidor* Dean, 2001: 75 fig. 13; 2004: 136.

###### Type locality.

Monterey Bay, California.

###### Type material.

Not seen.

###### Records.

California (Chamberlin 1918, Hartman 1947, 1969), Costa Rica, Gulf of Nicoya (Dean 2001, 2004).

###### Remarks.

*Leiochrides pallidor* it is a poorly known species and, until now, all descriptions have been incomplete. Chamberlin in his description only mentions that the species has 12 thoracic chaetigers, others features are not useful to characterize that species. Dean (2001) includes *Leiochrides* cf. *pallidor* for Costa Rica, which was characterized by the presence of 13 thoracic segments, the first one achaetous, and 12 complete chaetigers with capillary chaetae; however the illustration of the anterior end shows only 11 complete chaetigers with capillary chaetae, however, although the figure is wrong, the identification is correct (Dean pers. comm.)

#### Genus Mastobranchus

Eisig, 1887

Mastobranchus Eisig, 1887: 831

##### Type species.

*Mastobranchus trinchesii* Eisig, 1887

##### Mastobranchus variabilis

Ewing, 1984b

http://species-id.net/wiki/Mastobranchus_variabilis

Mastobranchus variabilis Ewing, 1984b: 793, fig. 1a–e; Dean 2001:76; 2004: 136.
Mastobranchus sp. A.Ewing 1984a: 14.35, Fig. 14.30a-f.
Mastobranchus ? *variabilis*Hernández-Alcántara and Solís-Weiss 1998: 710; 1999: 27.

###### Type locality.

Holotype (USNM), Florida, Tampa Bay, Gulf of México [27°36.5'N, 82°55.8'W], 12 m, clean sand, January 1980; 1 Paratype (USNM), Off Florida, St. Lucie Country, Hutchinson Island, Sta. 5 [27°22'22.08"N, 80°13'46"W], 10.3 m, Coll. D. Beaumariage, P. Camp and R. Gallagher, May 10 1972; and 1 Paratype (LACM-AHF), Sta. 723 [27°37.1'N, 82°55.1'W], 10 m, January 1980.

###### Type material.

Holotype (USNM 81993); 2 Paratypes (USNM 81994); 3 Paratypes (LACM- AHF 001369).

###### Material examined.

(6 specimens) Sinaloa: El Fuerte River, Stn. 51 [25°39.9'N, 109°30.6'W] (2 specimens), 49.5 m, March 20 1985, silty sands; Nayarit: María Madre Island Stn. 62C [21°38.2'N, 106°31.9'W] (4 specimens), 29.7 m, March 22 1985, fine sands Coll. PH-A.

###### Records.

USA: Alabama, Florida and North Carolina (Ewing 1984a–b), Costa Rica (Dean 2001, 2004).

###### Remarks.

*Mastobranchus variabilis* was described from the Gulf of México and later reported as *Mastobranchus*? *variabilis* by Hernández-Alcántara and Solís-Weiss (1998) from the Gulf of California. Upon analysis of the specimens from the Gulf of California we found instead that they agreed morphologically with *Neopseudocapitella brasiliensis* Rullier & Amoureux (1979) described from Brazil. The record of *Mastobranchus*? *variabilis* from the Mexican Pacific is therefore not considered valid.

#### Genus Mediomastus

Hartman, 1944

Mediomastus Hartman, 1944: 264

##### Type species.

*Mediomastus californiensis* Hartman, 1944

##### Mediomastus ambiseta

(Hartman, 1947)

http://species-id.net/wiki/Mediomastus_ambiseta

Capitita ambiseta Hartman, 1947: 409, Pl. 45, figs 1–4; 1969: 369, figs 1–4; Reish 1963: 428; 1968: 89.
Mediomastus ambiseta Warren et al., 1994: 234, figs 2a, 5d–f, 6a–f, 12; Blake 2000: 76, fig. 4.11; Dean 2001: 76; 2004: 136.

###### Type locality.

Newport Harbour Bay, Corona del Mar, Californa, USA [33°36'04"N, 117°52'48"W], June 2 1942, intertidal.

###### Type material.

Holotype (LACM-AHF POLY 1451-42, Poly 0449).

###### Material examined.

(41 specimens) Baja California: Todos Santos Bay, Stn. V-1 [116°39.4'N, 31°46"W] (5 specimens) 6 m, October 9 1979; [116°39.7'N, 31°45"W] (3 specimens), October 15 1989; San Quintín Bay, Stn. 13 [30°27'48"N, 115°57'46"W] (8 specimens), November 18 1982; Sinaloa: Off Ohuira Bay, Stn. B-2 [25°35'54"N, 109°20'12"W] (25 specimens), September 17 1989; Sinaloa: Topolobampo Bay (UANL-1613) [17°56'53"N, 102°5'9"W] (2 specimens), September 17 1989, Coll. JALG.

###### Records.

USA:California (Hartman 1947, Blake 2000 ; México: Baja California, San Quintín Bay (Reish 1963), Costa Rica (Dean 2001, 2004). México: Baja California, Sinaloa (present study).

##### Mediomastus californiensis

Hartman, 1944

http://species-id.net/wiki/Mediomastus_californiensis

Mediomastus californiensis Hartman, 1944: 264 Pl. 26, fig. 64; Hartman 1947: 408, Pl. 46, figs 3–4; 1963: 63; 1969: 387, figs 1–4; Day 1973: 99; Hutchings and Rainer 1979: 779; Ewing 1984a: 14.14, figs 14–10 a-c; Warren et al. 1994: 239, figs 2b, 7c–d, 9a–d, 12; de León González 1994: 62; Hernández-Alcántara and Solís-Weiss 1998: 710; Blake 2000: 78, fig. 4.12 a–d; Dean 2001: 78; 2004: 136.

###### Type locality.

California, Tomales Bay, USA [28°25'N, 123°W], June 8 1941, intertidal.

###### Type material.

Holotype (LACM-AHF POLY 0428).

###### Material examined.

(408 specimens) Baja California Sur: La Paz Bay, Ensenada de La Paz, Stn. 1 [24°06'50.3"N, 110°25'12.0"W] (10 specimens); Stn. 3 [24°07'09.9"N, 110°21'18.2"W] (1 specimen); Stn. 4 [24°08'29.9"N, 110°21'08.7"W] (20 specimens); Stn. 8 [24°09'50.7"N, 110°21'38.0"W] (8 specimens); Stn. 10 [24°09'55.1"N, 110°25'26.2"W] (42 specimens); Stn. 11 [24°08'28.9"N, 110°25'41.9"W] (7 specimens); Stn. 12 [24°07'37.3"N, 110°25'10.6"W] (4 specimens), 17 August 2005, 0.15 m; Stn. 1 (6 specimens); Stn. 2 [24°06'30.6"N, 110°24'05.1"W] (23 specimens); Stn. 3 (16 specimens); Stn. 6 [24°09'59.8"N, 110°19'37.5"W] (2 specimens); Stn. 8 (13 specimens); Stn. 11 (6 specimens) 5-7 m, 27 November 2005, Coll. DHV; Stn. 1 (17); Stn. 2 (59 specimens); Stn. 3 (51 specimens); Stn. 4 (2 specimens); Stn. 7 [24°09'00.0"N, 110°21'38.0"W] (1 specimen); Stn. 8 (6 specimens); Stn. 9 [24°10'13.9"N, 110°24'26.2"W] (3 specimens); Stn. 10 (9 specimens); Stn. 11 (1 specimen); Stn. 12 (1specimen) 30 m, 6 March 2006, Stn. 1 (5 specimens), Stn. 2 (17 specimens); Stn. 3 (3 specimens); Stn. 4 (4 specimens); Stn. 5 [24°08'53.4"N, 110°20'17.3"W] (1 specimen); Stn. 8 (7 specimens), Stn. 9 (4 specimens), Stn. 10 (15 specimens); Stn. 11 (4 specimens); Stn. 12 (1 specimen) 0.15 m, June 1 2006, Coll. DHV; Manglar Zacatecas (UANL-6425) [24°9'56.2"N, 110°25'55.6"W] (2 specimens), 24 June 2005; (UANL 6422) (15 specimens), 1 m, 1 August 2006; Enfermería Beach (UANL-6424) [24°13'55.6"N, 110°18'23.7"W] (2 specimens), 0.15 m, June 24 2005; El Conchalito Beach (UANL-6423) [24°08'23.4"N, 110°21'05.8"W] (20 specimens), 1 m, August 1 2006, Coll. JALG and MEGG.

###### Records.

USA: Oregon to California (Hartman 1944, 1947, 1963, 1969, Blake 2000); North Carolina (Day 1973); Florida (Ewing 1984a); Australia (Hutchings and Rainer 1979), California, Florida, North Carolina and British Columbia (Warren 1994), Costa Rica (Dean 2001, 2004). México: Baja California, Sonora (Hernández-Alcántara and Solís-Weiss 1993, 1998), Baja California Sur (de León-González 1994, present study).

##### Mediomastus setosus

Hartmann-Schöder, 1959

http://species-id.net/wiki/Mediomastus_setosus

Mediomastus setosus Hartmann-Schöder, 1959: 169, figs 173–177; 1959: 187; Molina-Lara and Vargas-Zamora 1995:202; Warren et al. 1994: 248, Rivera and Romero 2008: 19.

###### Type locality.

El Salvador, La Herradura, Estero Jaltepeque, [13°17'N, 89°02'W to 13°13'N, 88°54'W], February, 1955.

###### Type material.

Holotype (HZM P-19159). Not seen.

###### Records.

El Salvador, Jaltepeque Estuary (Hartmann-Schöder 1959, Molina-Lara and Vargas-Zamora 1995, Rivera and Romero 2008).

#### Genus Neoheteromastus

Hartman, 1960

Neoheteromastus Hartman, 1960:137

##### Type species.

*Neoheteromastus lineus* Hartman, 1960

##### Neoheteromastus lineus

Hartman, 1960

http://species-id.net/wiki/Neoheteromastus_lineus

Neoheteromastus lineus Hartman, 1960:137; Hartman 1963:63; Hartman 1969:389, figs 1–2; Fauchald 1972:243; Blake 2000: 87 fig. 4.16.
Notomastus sp. *fide* Bastida-Zavala, 1995:12.

###### Type locality.

San Nicolas, California, USA, 1609 m.

###### Type material.

Holotype (LACM-AHF POLY 0421).

###### Material examined.

(3 specimens) Baja California Sur:Cabo Pulmo-Los Frailes (1 specimen), Stn. CP-589-1 [23°24'27"N, 109°24'26"W], 4–7 m, May 7-8 1989, Coll. JRB-Z. South East of Gulf of California, Stn. Talud IV T-3–20 [24°27'N, 108°35'W] (1 specimen), 1,200-1,274 m, August 26 2000; Stn. Talud IV T-4–26 [24°56.3'N, 109°11.8'W] (1 specimen), 1,200–1,274 m, August 26 2000, Coll. Nuria Méndez-Ubach.

###### Records.

USA: California in deep slope and abyssal depths (Hartman 1960, 1963, 1969, Blake 2000). México: Baja California Sur, Cabo Pulmo-Los Frailes (Bastida-Zavala 1995 as *Notomastus* sp.), Guaymas basin, Gulf of California and off Tres Marías islands (Fauchald 1972). Sonora, Guaymas Basin Southern Trough, hydrothermal mounds (Blake 2000); Sinaloa (present study).

**Genus *Neomediomastus* Hartman, 1969**

##### Neomediomastus

Hartman, 1969

###### Type species.

*Mediomastus glabrus* Hartman, 1960

##### Neomediomastus glabrus

(Hartman, 1960)

http://species-id.net/wiki/Neomediomastus_glabrus

Mediomastus glabrus Hartman, 1960: 138.
Neomediomastus glabrus Hartman, 1969: 391, fig. 1; Fauchald 1972: 243; Warren et al. 1994: 250; Blake 2000: 88, fig. 4.17.

###### Type locality.

Southern California, Santa Catalina basin, Stn. 2850 [33°30'N, 118°35'W].

###### Type material.

Holotype (LACM-AHF POLY 0426).

###### Records.

USA: Basins off Southern California (Hartman 1960, 1969, Warren et al. 1994, Blake 2000); México: Baja California Sur, Nayarit in abyssal depths (Fauchald 1972).

#### Genus Neonotomastus

Fauchald, 1972

Neonotomastus Fauchald, 1972:245.

##### Type species.

*Neonotomastus glabrus* Fauchald, 1972

##### Neonotomastus glabrus

Fauchald, 1972

http://species-id.net/wiki/Neonotomastus_glabrus

Neonotomastus glabrus Fauchald, 1972: 245, Pl. 50, figs a–c.

###### Type locality.

Baja California Sur, Colorado Point, San Jose Island Stn. 11792 [25°20'00"N, 109°58'30"W], 405 m, November 24 1967.

###### Type material.

Holotype (LACM AHF POLY 1027).

###### Records.

México:Baja California Sur and Jalisco in abyssal depths (Fauchald 1972).

###### Remarks.

Fauchald (1972) described *Neonotomastus*, including the species *Neonotomastus glabrus*, but in his description of the genus he mentioned that the thorax had 10 segments with capillary chaetae. When examining the type material we found 11 thoracic segments with capillary chaetae. We consider this genus as valid. It is characterized by thoracic segments with capillaries, by the presence of a unirramous first chaetiger, a first abdominal chaetiger with notopodial capillary chaetae and the presence of neuropodial capillary chaetae and hooded hooks, the presence of capillary chaetae and hooded hooks in the notopodium and hooded hooks only in the neuropodium in the second abdominal segment.

#### Genus Neopseudocapitella

Rullier & Amoureux, 1979

Neopseudocapitella Rullier & Amoureux, 1979: 185 fig. 7 **Type species.***Neopseudocapitella brasiliensis* Rullier & Amoureux, 1979 http://species-id.net/wiki/Neopseudocapitella_brasiliensis
Neopseudocapitella brasiliensis Rullier & Amoureux, 1979: 185 fig. 7.
Mastobranchus ? variabilis *fide*Hernández-Alcántara and Solís-Weiss 1999: 27

##### Type locality.

Brazil, Stn. 49 [11°34'S, 37°22'W] 26 m, November 23 1961 intertidal in soft bottoms.

##### Type material.

Syntype (MNHN POLY TYPE 1301).

##### Material examined.

(3 specimens) Sonora: Puerto Peñasco, La Cholla Bay (UANL- 6519) [31°20'37.8"N, 113°38'01.7"W] (1 specimen), 1 m, August 7 2006, Coll. MEGG. Sinaloa, El Fuerte River, Stn. 51 [25°42.1'N, 109°30.6'W] (1 specimen), 49.5 m, March 20 1985; Nayarit, María Madre Island, Stn. 62C [21°38.2'N, 106°31.9'W] (1 specimen), March 22 1985, Coll. PHA.

##### Records.

Brazil (Rullier and Amoureux 1979); México: Sonora, Puerto Peñasco; Sinaloa, El Fuerte River; Nayarit, María Madre Island (present study).

##### Remarks.

The material of Hernández-Alcántara and Solís-Weiss (1998) identified as *Mastobranchus*? *variabilis* was analyzed and was found to be *Neopseudocapitella brasiliensis*. This record, and our collections from Puerto Peñasco, Sonora, constitutes the first record for this species in the Mexican Pacific.

#### Genus Notodasus

Fauchald, 1972

Notodasus Fauchald, 1972: 246, Pl. 51, figs a–c; García-Garza 2009: 101; García-Garza et al. 2009: 809.

##### Type species.

*Notodasus magnus* Fauchald, 1972.

##### Notodasus dexterae

Fauchald, 1973

http://species-id.net/wiki/Notodasus_dexterae

Notodasus dexterae Fauchald, 1973:27, fig. 2b–f; García-Garza 2009: 101; García-Garza et al. 2009: 809.

###### Type locality.

Panamá, Naos Island [8°53'N, 79°33'W], July 1969.

###### Type material.

Holotype (LACM-AHF POLY-2190) and 29 paratypes.

###### Records.

Panamá, Naos Island (Fauchald 1973; García-Garza et al. 2009).

##### Notodasus harrisae

García-Garza, Hernández-Valdez & de León-González, 2009

http://species-id.net/wiki/Notodasus_harrisae

Notodasus harrisae García-Garza, Hernández-Valdez & de León-González, 2009: 814, figs 3 A–D, 8C.

###### Type locality.

México: Baja California Sur, La Paz Bay, El Tesoro beach, [24°15'16.1"N, 110°18'55.4"W], 1 m, August 1, 2006, Coll. JALG and MEGG.

###### Type material.

Holotype (UANL-6510), 1 Paratype (LACM-AHF POLY 2211) , and 1 Paratype (MNHN 1506).

###### Material examined.

(46 specimens). Baja California: Los Angeles Bay, Municipal beach (UANL-6512) [28°57'10.8"N, 113°33'27.0"W] (1 specimen), 1 m, June 27 2005, Coll. JALG and MEGG; Baja California Sur: El Mogote beach, Stn. 89 (UANL-6505) [24°10'39"N, 110°22'75"W] (4 specimens), 0.50 m, October 2003, Coll. A. Chávez; Ensenada de La Paz, Stn. 2 (UANL-6506) [24°06'30.6"N, 110°24'05.1"W] (1 specimen), 0.15, cm November 27 2005; Stn. 5 (UANL-6507) [24°08'53.4"N, 110°20'17.3"W] (2 specimens), 0.15 cm, June 5 2006, Coll. DHV; El Requesón beach (UANL-6508) [26°44'39.1"N, 111°49'55.5"W] (1 specimen), 1 m, June 25 2005; El Quemadito beach, (UANL-6509) [26°45'33.1"N, 111°52'36.5"W] (1 specimen), 1 m June 26 2005, Colls. JALG and MEGG; Balandra beach (UANL-6511) [24°19'18.1"N, 110°19'29.4"W] (35 specimens), 1 m, August 1 2006, Coll. JALG and MEGG; Sinaloa: Mazatlán, Estero de Urías (ICMyL EMU-420) [23°12'N, 106°23'W] (1 specimen), 0 m, January 1979, Coll. A.R.

###### Records.

México: Baja California, Baja California Sur, Sinaloa (García-Garza et al. 2009).

##### Notodasus hartmanae

García-Garza, Hernández-Valdez & de León-González, 2009

http://species-id.net/wiki/Notodasus–hartmanae

Notodasus hartmanae García-Garza, Hernández-Valdez & de León-González, 2009: 815, figs 4A–D, 8D.

###### Type locality.

México:Paredón, Mar Muerto, Chiapas [16°03'26"N, 93°52'34"W], 0.5 m, April 14 2008, Coll. JALG,in compact mud, among numerous tubes of *Diopatra rhizophorae* Grube, with high content of organic matter.

###### Type material.

Holotype (UANL-6513), 6 Paratypes (UANL-6514), 3 Paratypes (LACM-AHF POLY 2212) and 3 Paratypes (MNHN1507).

###### Records.

México: Chiapas (García-Garza et al. 2009).

##### Notodasus kristiani

García-Garza, Hernández-Valdez & de León-González, 2009

http://species-id.net/wiki/Notodasus_kristiani

Notodasus kristiani García-Garza, Hernández-Valdez & de León-González, 2009: 817, figs 5A–D, 8E.

###### Type locality.

Sonora, Guaymas, Varadero beach [27°54'04.3"N, 110°52'07.7"W] 1 m, July 01 2005, Coll. MEGG and JALG.

###### Type material.

Holotype (UANL-6515), 20 Paratypes (UANL-6517), 2 Paratypes (LACM-AHF POLY 2213) and 1 Paratype (MNNH 1508).

###### Material examined.

(2 specimens) Baja California: Los Angeles Bay, Municipal beach (ECOSUR) [28°56'32.3"N, 113°33'57.2"W] (1 specimen), 1 m, May 24 1986, Coll. P. Sánchez and E. Espinosa; Baja California Sur: Santa Marina Bay, Estero Rancho Nuevo (UANL-6515) [24°19'15"N, 111°25'05"W] (1 specimen), 3 m, June 21 1998, Coll. JALG.

###### Records.

México:Baja California, in mud pockets between *Mytilus edulis* beds, Baja California Sur, in soft sediments retained into Nextier boxes, Sonora, in mud with high content of organic matter (García-Garza et al. 2009).

##### Notodasus magnus

Fauchald, 1972

http://species-id.net/wiki/Notodasus_magnus

Notodasus magnus Fauchald, 1972: 246, Pl. 51 figs a–c; García-Garza et al. 2009: 818, figs 6A–D, 8F.

###### Type locality.

Isla Carmen, Punta Arena, Gulf of California [25°46'00"N, 111°15'00"W; 25°49'40"N, 111°15'30"W], 29-35 m, March 18 1949, in silts with sand, mud and cobbles.

###### Type material.

Holotype **(**LACM-AHF POLY 1031).

###### Records.

México: Baja California Sur, Isla Carmen (Fauchald 1972, García-Garza et al. 2009), only known.

#### Genus Notomastus

Sars, 1851

Notomastus Sars, 1851:199

##### Type species.

*Notomastus latericeus* Sars, 1851.

##### Notomastus aberans

Day, 1957

http://species-id.net/wiki/Notomastus_aberans

Notomastus aberans Day, 1957: 105, fig. 7 a–b; Day 1967: 599, fig. 28.1 m–q; de León González 1994: 63 fig. 11 a.

###### Type locality.

South Africa, Kosi Bay

###### Type material.

Holotype (ANO3 1961.16. 75–76)

###### Material examined.

(1 specimen) Western coast of Baja California Sur, Stn. A-3 (UANL-0038) [24°11.4'N, 111°23.5'W] (1 specimen), 74 m, June 17 1987.

###### Records.

South Africa (Day 1957, 1967).

###### Remarks.

*Notomastus aberans* was described from South Africa and de León-González (1994) reported it for the western coast of the Baja California Peninsula. Examination of the Mexican material, found that this was *Notomastus polyodon* Gallardo, 1968 described from Vietnam. This was corroborated after the revision of the holotype of that species. The Mexican specimens as well as the type material of *Notomastus polyodon* have a mid-dorsal lobe between the thoracic notopodia on chaetiger 8 to 11 and the abdominal notopodia and neuropodia have expanded edges appearing as triangular lobes. Furthermore, methyl green staining patterns are similar in both specimens, with the prostomium and first three segments staining light green, segments 4 to 8 intensely green, segments 9 to 11 with a wide prechaetal dark green band moderately staining, abdominal segments, and two intensely staining lateral bands to the end of the fragment.

##### Notomastus abyssalis

Fauchald, 1972

http://species-id.net/wiki/Notomastus_abyssalis

Notomastus abyssalis Fauchald 1972: 248, Pl. 51, figs d–g.

###### Type locality.

Baja California Sur: Punta Colorado, San José Island, Stn.11788 [25°21'00"N, 110°05'00"W], November 24 1967, 393m depth, only known for deep waters, from 375 to 481m, in silts with fine sand.

###### Type material.

Holotype (LACM-AHF POLY 1012).

###### Material examined.

(10 specimens)Baja California Sur: Cabo Falso, Stn. 13774 [22°35'00"N, 109°35'00"W] (1 specimen), 438 m, January 22 1970; Stn. 13775 [22°34'00"N, 109°35'45"W] (1 specimen), 441 m, January 22 1970; Punta Colorado, San Jose Island, Stn. 11788 [25°21'00"N, 110°05'00"W] (1 specimen), 393 m, November 23 1967; Stn. 11792 [25°20'00"N, 109°58'30"W] (1 specimen), 405 m, November 23 1967; Stn. 11793 [25°20'00"N, 109°59'00"W] (2 specimens) 408 m November 23 1967; Sinaloa: Mazatlán, Crestón Island,Stn. 11761 [23°05'00"N, 107°59'20"W] (2 specimens) 405 m, November 14 1967; Jalisco: Cabo Corrientes Stn. 13752 [19°41'08"N, 105°53'30"W] (1 specimen) 375 m, January 18 1967; Nayarit: San Juanito Island, Tres Marías Island Stn. 13765 [21°37'00"N, 106°57'30"W] (1 specimen), 481 m, January 20 1970.

###### Records.

México:Baja California Sur, Southern and central part of the Gulf of California, and Nayarit, Tres Marías Islands (Fauchald 1972).

##### Notomastus americanus

Day, 1973

http://species-id.net/wiki/Notomastus_americanus

Notomastus americanus Day, 1973: 100, fig. 131; Ewing 1984a: 14.31, figs 14.25, 14.26 a–d; Hernández-Alcántara and Solís-Weiss 1993: 1034; 1998: 710; 1999: 27.

###### Type locality.

North Carolina, Beaufort, June 4 1965.

###### Type material.

Holtype (USNM 43118) and 14 Paratypes (USNM–43119).

###### Material examined.

(57 specimens),Baja California: Punta Willard, Stn. 34 [30 11.5N, 114 31.7'W] (1 specimen), 32.2 m, 15 March 1985; Rocks Consag, Stn. 39 [30°59.4"N, 114°04.1"W] (12 specimens), 106.4 m, March 16 1985, silty sand; Baja California Sur: Santa Inés Bay, Stn. 49 [26°59.6'N, 111°50.4'W] (3 specimens), 100 m, March 19 198; Sonora: Punta Arboleada, Stn. 14 [26°46.6'N, 110°06.7'W] (8 specimens), 92 m, March 12 1985; Stn. 15 [26°51.1'N, 110°06.5'W] (13 specimens), 48.8 m, March 12 1985; Cabo Tepoca, Stn. 44 [30°02.4'N, 112°55.4'W] (9 specimens), 100 m, March 17 1985, Sinaloa: Santa María Bay, Sta. 3 [25°02.4'N, 108°31.7'W] (1 specimen), 32 m, March 10 1985; Stn. 4 [24°56.9'N, 108°31.7'W] (4 specimens), 79m March 10 1985; Río Fuerte, Stn. 50, [25°46.8'N, 109°35.4'W] (4 specimens), 97 m March 20 1985; Stn. 51 [25°42.1'N, 109°30.6'W] (2 specimens), 49.5 m March 20 1985, Coll. PHA.

###### Records.

USA: North Carolina (Day 1973, Ewing 1984).

###### Remarks.

*Notomastus americanus* Day 1973 was described from North Carolina and reported by (Hernández-Alcántara and Solís-Weiss (1993, 1998, 1999) from the Gulf of California. Following examination of the Mexican specimens, they were found to belong instead to the species *Notomastus hemipodus* Hartman, 1945.

##### Notomastus angelicae

Hernández-Alcántara & Solís-Weiss, 1998

http://species-id.net/wiki/Notomastus_angelicae

Notomastus angelicae Hernández-Alcántara & Solís-Weiss, 1998:713, figs 1 a–f, 2–3.

###### Type locality.

Sinaloa, West Río Fuerte [25°39'54"N, 109°28'36"W], 28.6 m, March 1985.Silt with fine sand.

###### Type material.

Holotype (USNM 180697), 5 Paratypes (USNM 180698) and 5 Paratypes (LACM-AHF-POLY-1902).

###### Material examined.

(104 specimens) Baja Clifornia: Punta San Marcial Stn. 10 [25°58.6'N, 111°06.9'W] (4 specimens), 39 m, March 11 1985; Punta Willard, Stn. 30 [30°11.59'N, 114°31.7'W] (1 specimen), 32.2 m, 15 March 1985,; Baja California Sur: western coast, Stn. E-14 [25°38'12"N, 112°23'18"W] (2 specimens), 80 m, Coll. JALG; Santa Inés Bay, Stn. 49 [26°59.6'N, 111°50.4'W] (3 specimens), 100 m, 19 March 1985; Stn. 49B [26°59.4' N, 111°53.5'W] 68 m, 19 March 1985; Sonora: Punta Arboleada, Stn. 14 [26°46.6'N, 110°06.7'W] (1 specimen), 92 m, March 12 1985; Cabo Tepoca, Stn. 44 [30°02.4'N, 112°55.4'W] (9 specimens), 100 m, March 17 1985; Sinaloa: Santa María Bay, Stn. 4 [24°56.9'N, 108°31.7'W] (4 specimens), 79 m, March 10 1985; Stn. 5 [24°54.6'N, 108°45.3'W] (2 specimens), 120 m, 10 March 1985; Río Fuerte, Stn. 50 [25°46.8'N, 109°35.4'W], (9 specimens), 97m; Stn. 51 [25°42.1'N, 109°30.6'W] (1 specimen), 49.5 m, March 20 1985; Nayarit: Islas Marías Stn. 62C, [21°38.2'N, 106°31.9'W] (56 specimens), 29.7 m, March 22 1985, Coll. PHA.

###### Records.

México:Baja California Sur, Sonora, Sinaloa (Hernández-Alcántara and Solís-Weiss 1998).

##### Notomastus cinctus

Fauchald, 1972

http://species-id.net/wiki/Notomastus–cinctus

Notomastus cinctus Fauchald, 1972: 250, Pl. 50, figs d–h.

###### Type locality.

Baja California Sur, Cabo Falso [22°34'00"N, 109°32'30"W], 1,450 m January 22 1970.

###### Type material.

Holotype (LACM AHF POLY 1026).

###### Records

.México: Baja California Sur, Cabo Falso; Guerrero,Zihuatanejo Bay, Nayarit, in 1,200 to 1,450 m (Fauchald 1972).

##### Notomastus hemipodus

Hartman, 1945

http://species-id.net/wiki/Notomastus_hemipodus

Notomastus (Clistomastus) hemipodus Hartman, 1945:38; 1947: 424, Pl. 48, figs 1–5; 1951: 103, Pl. 24, figs 1–3; 1969: 393, figs1–5.
Notomastus hemipodus Day, 1973: 100; Ewing 1984a: 14.28, figs 14.23, 14.24 a–d; Blake 2000: 81, figs 4.13 a–e; Dean 2001: 79, 2004: 136.
Notomastus near hemipodus Green, 2002: 297, fig. 17 a–k.
Notomastus (Clistomastus) tenuis fide Fauchald, 1972: 248.
Notomastus americanus *fide*Hernández-Alcántara and Solís-Weiss 1993: 1034; 1999: 27; 2003: 4.
Notomastus tenuis fide Hernández-Alcántara and Solís Weiss 1999: 27; 2003: 4.

###### Type locality.

North Carolina, Beaufort, June 15-18 1940, intertidal fine sands and mud.

###### Type material.

Holotype (LACM-AHF POLY 0414), 1 Paratype (LACM-AHF POLY 1697), 1 Paratype (LACM-AHF POLY 0415), 2 Paratypes (LACM-AHF POLY 1701) and 5 Paratypes (LACM-AHF POLY 1709).

###### Material examined.

(57 specimens) Baja California: Consag Rocks, Stn. 38 [31°08.3N, 114°13.3"W] (4 specimens), 71.9 m, 16 March 1985; Stn. 39 [30°59.4"N, 114°04.1"W] (5 specimens), 106.4 m, March 16 1985; Santa Inés Bay, Stn. 49C [26°59.2"N, 111°58.3"W] (5 specimens), Coll. PHA; Baja California Sur: West Coast (UANL- 6486), Stn. 25 BIP II (1 specimen), October 6 1998, Colls. Eduardo Balart and EAS; (UANL 6485) (2 specimens), Rancho Bueno, Santa Marina Bay [24°08'23.4"N, 110°21'05.8"W], 1 m, January 21 1999, Coll. JALG; La Paz Bay, Ensenada de La Paz (UANL-6482), Stn. 12 [24°07'37.3"N, 110°25'10.6"W] (1 specimen), 0.15 m, November 27 2005, Coll. DHV; (UANL-6484), Stn. 10-A [24°09'55.1"N, 110°25'39.6"W] (1 specimen), 1 m, November 27 2005, Coll. DVH; El Conchalito beach (UANL-6481) [24°08'23.4 N, 110°21'05.8"W] (2 specimens), 1 m, June 24 2005; Concepción Bay, Los Cocos beach (UANL-6483) [26°44'39.1 N, 111°53'55.4"W] (2 specimens), 1 m, June 25 2005; Santispac mangrove (UANL-6487) [26°45'43.2N, 111°53'31.0"W] (6 specimens), 1 m, August 3 2006, Colls. JALG and MEGG; Sonora: Punta Arboleda Stn. 15 [26°51.1"N, 110°06.5"W] (13 specimens), 49.8 m, March 12 1985; Estero Tastiota Stn. 48 [28°16.4"N, 111°36.6"W] (5 specimen), 36.9 m, 18 March 1985; Sinaloa: Santa María Bay, Stn.3 [25°02.4"N, 108°31.7"W] (1 specimen), 32 m, March 10 1985; Stn. 5 [24°54.6"N, 108°45.3"W] (5 specimens), 79 m, March 10 1985; El Fuerte, River Stn. 52 [25°39.9"N, 109°28.6"W] (5 specimens), 28.6 m, March 20 1985, Coll. PHA.

###### Records.

USA: Beaufort, North Carolina (Hartman 1947), North Gulf of México (Hartman 1951, Ewing 1984a), California (Hartman 1969, Blake 2000), Costa Rica (Dean 2001, 2004), Andaman Sea as *Notomastus near hemipodus* (Green, 2002), México: Baja California, Baja California Sur, Sonora, Sinaloa (Hernández Alcántara and Solís Weiss 1993, 1999, 2003 as *Notomastus americanus* and *Notomastus tenuis*, present study).

##### Notomastus latericeus

Sars, 1851

http://species-id.net/wiki/Notomastus_latericeus

Notomastus latericeus Sars, 1851:199; Fauvel 1927:143, fig. 49a–h; 1953: 364, fig. 189 a–h; Wesenberg-Lund 1949: 336; Day 1961: 519; 1967: 599, fig. 28.2a–d; Gallardo 1968: 120, Pl. 53 fig. 13; Thomassin 1970: 83, fig. 8a-c; Ewing 1984a: 14.24 figs 14.17, 14.18; Hernández-Alcántara and Solís-Weiss 1993: 1034; 1998: 711; 1999: 27.

###### Type locality.

Norway (Atlantic Ocean)

###### Type material.

Not seen

###### Material examined.

(15 specimens) Baja California: Stn. 30 [11.59'N, 114°31.7'W] (3 specimens), 32.2 m, March 1985; Punta Willard, Stn. 34 [30°11.5'N, 114°31.7'W] (1 specimen), 32.4 m, March 15 1985; Baja California Sur: Santa Inés Bay, Stn. 49B [26°59.4'N, 111°53.5'W] (1 specimen), 68 m, March 19 1985; Sonora: Punta Arboleada, Stn. 14 [26°46.6'N, 110°06.7'W] (1 specimen), 92 m; Cabo Tepoca, Stn. 44 [30°02.4'N, 112°55.4'W] (5 specimens), 104.1 m, March 17 1985; Sinaloa: Santa María Bay, Stn.4 [24°56.9'N, 108°31.7'W] (4 specimens), 32 m, Coll. PHA.

###### Records.

Norway (Sars 1851). France (Fauvel 1927). Gulf of Iran (Wesenberg-Lund 1949). South Africa, Mozambique, Madagascar (Day 1961, 1967, Thomassin 1970). Viet Nam (Gallardo 1968). Northern Gulf of México (Ewing 1984a).

###### Remarks.

*Notomastus latericeus* Sars, 1851 was described from Norway and has been reported widely around the world. Hernández-Alcántara and Solís-Weiss (1993, 1998 and 1999) reported it from the Gulf of California but following examination of their material it was found that they are similar to the type material of *Notomastus magnus* Hartman, 1947.

##### Notomastus lineatus

Claparède, 1870

http://species-id.net/wiki/Notomastus_lineatus

Notomastus lineatus Claparède, 1870: 18, Pl. 17 fig. 4; Fauvel 1927: 145, fig. 51a–i; Berkeley and Berkeley 1932: 674; Ewing 1984a: 14.24, fig. 14.18a–e; Bastida-Zavala 1993: 22; Hernández-Alcántara and Solís-Weiss 1998: 712; 1999: 27 (*partim*), Dean 2001: 80; 2004: 137.
Notomastus (Clistomastus) lineatus Hartman, 1947: 419, Pl. 46, figs 1–2; Hartman 1969: 395 figs 1–5.

###### Type locality.

Gulf of Naples (Mediterranean Sea).

###### Type material.

Not seen

###### Material examined.

(7 specimens), Baja California: Cabo San Miguel, Sta. 19 [28°10.4'N, 112°48.1'W] (1 specimen), 30.4 m March 13 1985; Baja California Sur: Banco Gorda, Sta. 55 [23°08.7'N, 119°28.3'W] (1 specimen), 32.5m, March 21 1985; Santa Inés Bay, Sta. 49C [26°59.2'N, 111°58.3'W] (1 specimen), 28.9 m, March 19 1985; Punta San Marcial, Sta. 8 [25°33.4'N, 110°59.8'W] (1 specimen), 52 m, March 11 1985; Sta. 9 [25°47.8'N, 110°03.8'W] (1 specimen), 77.5 m, March 11 1985, Coll. PHA; La Paz Bay, Caimancito beach, Stn. C37 (2 specimens), November 29 1986, Coll. JRB-Z

###### Records.

Gulf of Naples, Mediterranean Sea (Claparède 1870), France (Fauvel 1927), Canada (Berkeley & Berkeley 1932), California (Hartman 1947, 1969), Northern Gulf of México (Ewing 1984), Costa Rica (Dean 2001, 2004).

###### Remarks.

The specimens reported by Bastida-Zavala (1993) as *Notomastus lineatus* from Baja California Sur are in fact *Dasybranchus parplatyceps* Kudenov 1975. Hernández-Alcántara and Solís-Weiss (1998) reported *Notomastus lineatus* for Baja California and Baja California Sur, however, examination of their specimens determined them to be a mixture of *Notomastus magnus*, *Notomastus polyodon*, *Notomastus cinctus* and *Notomastus angelicae*. The report of *Notomastus lineatus* are doubtful for western México although the species has been reported from both California (USA) Blake (2000) and the Pacific Costa Rica (Dean, 2001).

##### Notomastus magnus

Hartman, 1947

http://species-id.net/wiki/Notomastus_magnus

Dasybranchus giganteus : Moore 1909: 279.
Notomastus giganteus : Berkeley and Berkeley 1941: 48.
Notomastus magnus : Hartman 1947: 412, Pl.50, figs 1–6; 1961:35; 1963:63; 1969:401, figs 1–6; Reish 1963: 429; Blake 2000: 83, fig. 4.14.

###### Type locality.

USA: California, Tomales Bay.

###### Type material.

(1)Paratype (LACM-POLY 0413) and 1 Paratype (LACM-POLY 2217).

###### Material examined.

(6 specimens) Baja California Sur: Magdalena Bay [25°36'8"N, 112°0'8"W] (1 specimen), 1.5 m, December 6 1996, Coll. JALG; Sonora: Puerto Peñasco, La Cholla Bay [31°20'37.8"N, 113°38'01.7"W] (5 specimens), 1.5 m, August 7 2006, Coll. JALG and MEGG.

###### Records.

USA: California (Moore 1909 as *Dasybranchus giganteus*; Berkeley & Berkeley 1941 as *Notomastus giganteus*; Hartman 1947, 1961, 1963, 1969, Blake 2000) México: Western coast of Baja California Sur and Northern Gulf of California, Sonora (present study)

##### Notomastus polyodon

Gallardo, 1968

http://species-id.net/wiki/Notomastus_polyodon

Notomastus polyodon Gallardo, 1968: 120, Pl. 56 fig. 1– 4; Green 2002: 303, fig. 20.
Notomastus aberrans ? *fide*de León-González 1994: 63.

###### Type locality.

South Viet Nam, Nha Trang Bay, Stn. 26411. Soft bottoms, intertidal zone.

###### Type material.

Holotype (LACM-AHF POLY 0301)

###### Material examined.

(59 specimens) Baja California Sur: west coast, Stn.A-3 (UANL-0038) [24°11.4'N, 111°23.5'W] (1 specimen), 74 m, June 17 1987, Coll. JALG.Baja California Sur: La Paz Bay, Caleritas beach (1 specimen), Stn. Ca-29 N-8, November 29 1986, Coll. JRB-Z; Ensenada de La Paz (UANL-6521), Stn. 1 [24°06'50.3"N, 110°25'12.0"W] (1 specimen); (UANL-6522), Stn. 4 [24°08'29.9"N, 110°21'08.7"W] (2 specimen); (UANL-6523), Stn. 6 [24°09'59.8"N, 110°19'37.5"W] (1 specimen); (UANL 6524), Stn. 7 [24°09'00.0"N, 110°21'38.0"W) (1 specimen); (UANL-6525), Stn. 8 [24°09'50.7"N, 110°23'35.9"W] (3 specimens); (UANL-6526), Stn. 9 [24°10'13.9"N, 110°24'26.2"W] (3 specimens); (UANL-6527), Stn. 10 [24°09'55.1"N, 110°25'39.6"W] (7 specimens); (UANL-6528), Stn. 11 [24°08'28.9"N, 110°25'41.9"W] (2 specimens), 0.15 m, Coll. DHV; Balandra beach (UANL-6529) [24°19'18.1"N, 110°19'29.4"W] (3 specimens), August 1 2006; Concepción Bay, Santispac beach (UANL 6530) [26°45'45.3"N, 111°53'30.0"W] (1 specimen), 1 m, June 26 2005, Coll. MEGG and JALG; El Quemadito beach, (UANL-6531) [26°45'33.1"N, 111°52'36.5"W] (14 specimens), 1 m, June 26 2005, Coll. MEGG and JALG; Sonora: Puerto Peñasco, La Cholla Bay (UANL-6532) [31°20'37.8"N, 113°38'01.7"W] (2 specimens), 1 m, June 29 2005. Sinaloa: Mazatlán, Punta Cerritos beach (UANL -6533) [23°18'28.6"N, 106°27'21.3"W] (21 specimens), 0.50 m, May 22 2006, Coll. JALG and MEGG; Estero de Urías (UANL-6534)[23°12'00"N, 106°23'00"W] (13 specimens), 0.50 m, January 1979, Coll. A.R.

###### Records.

South Viet Nam (Gallardo 1968). Andaman Sea (Green 2002). México:Western Coast of Baja California Sur (de León-González 1994); Baja California (Gulf of California), Sonora, Sinaloa (material reported in this study).

###### Remarks.

*Notomastus polyodon* was previously known only from the western Pacific, this is the first formal record from the eastern Pacific.

##### Notomastus precocis

Hartman, 1960

http://species-id.net/wiki/Notomastus_precocis

Notomastus precocis Hartman, 1960: 139; 1969: 403; Fauchald 1972: 251.

###### Type locality.

USA: Santa Catalina Gulf, 1400-2000m.

###### Type material.

Holotype (LACM-AHF POLY 0416).

###### Material examined.

(6 specimens)Baja California Sur: Natividad Island Stn. 7358 [27°35'45"N, 115°08'30"W to 27°32'15"N, 115°05'00"W] (3 specimens), 182–201 m, April 21 1961; Jalisco: Cabo Corrientes, Stn. 13754 [19°41'15"N, 105°53'00"W] (2 specimens), 365 m, January 18, 1970; Nayarit: Punta Piedras, Stn. 13767 [21°54'30"N, 106°50'00"W] (1specimen), 246 m January 20 1970.

###### Records.

USA: California (Hartman 1960). México: Baja California, Jalisco, Nayarit in the slope zone (Fauchald 1972).

##### Notomastus sonorae

Kudenov, 1975

http://species-id.net/wiki/Notomastus_sonorae

Notomastus (Notomastus) sonorae Kudenov, 1975: 221, figs 35–39.

###### Type locality.

Sonora, La Cholla Bay, Puerto Peñasco, march 28 1971. Intertidal, in sediments with fine sand.

###### Type material.

Holotype (LACM-AHF POLY 1113).

###### Material examined.

(11 specimens) Sonora: Puerto Peñasco, La Cholla Bay (UANL- 6536) [31°20'37.8"N, 113°38'01.7"W], 1 m, June 29 2005, Coll. JALG and MEGG.

###### Records.

México: Sonora, (Kudenov 1975).

##### Notomastus tenuis

Moore, 1909

http://species-id.net/wiki/Notomastus_tenuis

Notomastus tenuis Moore, 1909: 277, Pl. 9, fig. 55; Hartman 1947: 420; Berkeley and Berkeley 1952: 103; Blake 2000: 85, figs 4.15; Dean 2001: 81; 2004: 147; Hernández-Alcántara and Solís-Weiss 1998: 712; 2003: 4.
Eisigella tenuis Berkeley & Berkeley, 1942: 198.
Notomastus (Clistomastus) tenuis Hartman 1969: 397, figs 1–5; Reish 1968:89; Kudenov 1975: 220; Calderón-Aguilera and Jorajuria-Corbo 1986: 55, fig. 8 a–c.
Notomastus? tenuis Ewing, 1984a: 14.26.

###### Type locality.

San Diego, California, USA, 1902-1903, intertidal. Coll. E. C. Starks.

###### Type material.

Holotype (CAS-019718).

###### Material examined.

(80 specimens)California: Anaheim Slough Collections: (LACM-AHF), Stn. 903-38 (7 specimens), December 5 1938; (5 specimens), Stn. 905–38, December 7 1938; (2 specimens), Mission Bay, Stn. 1211–38; Baja California: Las Animas Island (LACM-AHF), Stn. F3893-11831 [28°41'30"N, 113°02'00"W] (1 specimen), 207 m, December 1 1967, Coll. VELERO IV; Baja California Sur: (LACM-AHF), Cabo Falso, Stn. F4285-13774 [22°35'00"N, 109°35'00"W] (1 specimen), 426.72 m, January 22 1970, Coll. VELERO IV; Sonora, Punta Arboleda (ICMyL-UNAM), Stn. 15 [26°51.1'N, 110°06.5'W] (13 specimens), 49.8 m, March 12 1985, Coll. PHA; Estero Tastiota (ICMyL-UNAM), Stn. 48 [28°16.4'N, 111°36.6'W] (5 specimens), 60.2 m, March 18 1985, Coll. PHA; Sinaloa: El Fuerte River (ICMyL-UNAM), Stn. 52 [25°39.9'N, 109°28.6'W] (5 specimens), 28.6 m, March 20 1985, Coll. PHA; Santa María Bay (ICMyL-UNAM), Stn. 3 [25°02.4'N, 108°31.7'W] (1 specimen), 32 m March 10 1985 ; Stn. 05 [24°54.6'N, 108°45.3'W] (5 specimens), 120 m, March 10 1985; Jalisco: Cabo Corrientes (LACM-AHF), Stn. F4205-13756 [19°51'30"N, 105°58'00"W] (1 specimen), 426.72 m January 18 1970, Coll. VELERO IV. Nayarit: Punta Piedras, San Juanito Island (LACM-AHF), Stn. F4296-13768 [21°53'00"N, 106°50'00"W] (1 specimen), 256 m January 20 1970, Coll. VELERO IV; North Rock Consag (ICMyL-UNAM), Stn. 38 [31°08.3'N, 114°13.3'W] (4 specimens), 71.9 m March 16 1985; Stn. 39 [30°59.4'N, 114°04.1'W] (12 specimens), 106.4 m, March 16 1985, Coll. PHA.

###### Records.

USA: California (Moore 1909, Hartaman 1947, Berkeley and Berkeley 1952, Blake 2000), Costa Rica (Dean 2001, 2004).

###### Remarks.

*Notomastus tenuis* was described from California by Moore (1909). Fauchald (1972) reported it from Baja California, Baja California Sur, Nayarit and Jalisco while Hernández-Alcántara and Solís-Weiss (1998) reported it from the Gulf of California. Examination of specimens of both author's collections indicated that they were actually *Notomastus hemipodus* Hartman, 1945. The report by Calderón-Aguilera and Jorajuria-Corbo (1986) for San Quintín Bay seems to be incorrect, as the description and illustrations do not correspond to *Notomastus tenuis*. The records of *Notomastus tenuis* from western of México are therefore considered doubtful.

#### Genus Rashgua

Wesenberg-Lund, 1949

Rashgua Wesenberg-Lund, 1949: 336

##### Type species.

*Rashgua rubrocincta* Wesenberg-Lund, 1949

##### Rashgua lobatus

(Hartman, 1947)

http://species-id.net/wiki/Rashgua_lobatus

Notomastus lobatus Hartman, 1947: 415, Pl. 51, figs 1–5; 1969: 399, figs 1–5; Ewing 1984a: 22, fig. 18.
Rashgua near lobatus Green, 2002: 309, fig. 22 a–g.

###### Type locality.

Baja California, Espíritu Santo Island, México, Stn. 1107-40, 6–10 m,intertidal in soft bottoms.

###### Type material.

Holotype (LACM-AHF POLY 1107–40), 1 Paratype (LACM-AHF POLY 0411) and 1 Paratype (LACM-AHF POLY 1069-40).

###### Material examined.

(173 specimens) Baja California Sur: La Paz Bay, Caimancito beach, Stn. C-37 [24°08'23.4"N, 110°18'00"W] (8 specimens), 1.5 m, February 29 1986, Coll. Héctor Salaices-Polanco; Ensenada de la Paz (UANL-6554), Stn. 3 [24°07'09.9"N, 110°21'18.2"W] (12 specimens), 0.20 m, November 2005; (UANL-6555), Stn. 2 [24°06'30.6"N, 110°24'05.1"W] (52 specimens), 0.20 m, March 6 2006; (UANL-6556), Stn. 2 (106 specimens), 27 m, 1 June 2006, Coll. DHV; Sonora: Guaymas, Varadero beach (UANL-6557) [27°54'04.3"N, 110°52'07.7"W] (3 specimens), 1 m, July 1 2005, Coll. JALG and MEGG.

###### Records.

USA: Gulf of México, Mobile Bay, Mississippi, North Carolina (Ewing 1984a), Thailand (Green 2002 as *Rashgua near lobatus*). México: Baja California (Hartman 1947, 1969), Baja California Sur, Sonora (material reported in this study).

#### Genus Scyphoproctus

Gravier, 1904

Scyphoproctus Gravier, 1904:557.

##### Type species.

*Scyphoproctus djiboutiensis* Gravier, 1904.

##### Scyphoproctus oculatus

Reish, 1959

http://species-id.net/wiki/Scyphoproctus_oculatus

Scyphoproctus oculatus Reish, 1959: 78; Reish 1963: 429; Hartman 1969: 405; Salazar-Vallejo 1991: 85.

###### Type locality.

California, New Port Bay, intertidal, in soft bottoms with fine sand and shells fragments.

###### Type material.

Holotype (LACM-AHF POLY 11108, POLY 0481) and 1 Paratype (BMNH AN03 1959.12.18.1).

###### Material examined.

(13 specimens) Baja California Sur: La Paz Bay, San Gabriel (UANL -6559) [24°18'44.3"N, 110°20'10.7"W] (2 specimens), August 31 2004; Concepción Bay, Santispac beach (UANL-6561) [26°45'45.3"N, 111°53'30.0"W] (3 specimens), 0.50 m, June 25 2005, Colls. JALG and MEGG.

###### Records.

USA: Southern California (Reish 1959, Hartman 1965, 1969). México: Baja California, San Quintín Bay (Reish 1963), Gulf of California, Rasa Island (Salazar-Vallejo 1991); Baja California Sur (material reported in this study).

## Conclusion

In this study we first found 43 species of Capitellidae from Eastern Tropical Pacific belonging to 19 genera. Following analysis of type and non-type material we conclude that this list should be reduced to 32 valid species in 17 genera. Of these, 14 species were originally described from the Eastern Tropical Pacific.

The species *Capitella capitata* Fabricius 1780, *Dasybranchus glabrus* Moore, 1909, *Dasybranchus lumbricoides* Grube 1878, *Notomastus lineatus* Claparède 1870 and *Notomastus tenuis* Moore 1909 are considered as doubtful records. Also, the species *Decamastus gracilis* Hartman 1963, *Mastobranchus variabilis* Edwing 1984, *Notomastus aberans* Day 1957, *Notomastus americanus* Day 1973, *Notomastus latericeus* Sars 1851, are determined to have been erroneously reported for the Eastern Tropical Pacific. In the case of *Decamastus nudus*Thomassin, 1970, we consider that the specimens reported from Gulf of California by Henández-Alcántara and Solís-Weiss belong to an undescribed species.

*Amastigos acutus* (Piltz 1977), *Mediomastus ambiseta* Hartman 1947, *Neopseudocapitella brasiliensis* (Rullier & Amoreux 1979) and *Notomastus polyodon* (Gallardo 1968), are listed as new records for the Eastern Tropical Pacific.

## Supplementary Material

XML Treatment for Amastigos

XML Treatment for Anotomastus

XML Treatment for Capitella

XML Treatment for Dasybranchethus

XML Treatment for Dasybranchus

XML Treatment for Heteromastus

XML Treatment for Leiocapitella

XML Treatment for Leiochrides

XML Treatment for Mastobranchus

XML Treatment for Mediomastus

XML Treatment for Neoheteromastus

XML Treatment for Neonotomastus

XML Treatment for Neopseudocapitella

XML Treatment for Notodasus

XML Treatment for Notomastus

XML Treatment for Rashgua

XML Treatment for Scyphoproctus
